# Bi-allelic variants in *POPDC2* cause an autosomal recessive syndrome presenting with cardiac conduction defects and hypertrophic cardiomyopathy

**DOI:** 10.1016/j.ajhg.2025.04.016

**Published:** 2025-05-22

**Authors:** Michele Nicastro, Alexa M.C. Vermeer, Pieter G. Postema, Rafik Tadros, Forrest Z. Bowling, Hildur M. Aegisdottir, Vinicius Tragante, Lukas Mach, Alex V. Postma, Elisabeth M. Lodder, Karel van Duijvenboden, Rob Zwart, Leander Beekman, Lingshuang Wu, Sean J. Jurgens, Paul A. van der Zwaag, Mariëlle Alders, Mona Allouba, Yasmine Aguib, J. Luis Santome, David de Una, Lorenzo Monserrat, Antonio M.A. Miranda, Kazumasa Kanemaru, James Cranley, Ingeborg E. van Zeggeren, Eleonora M.A. Aronica, Michela Ripolone, Simona Zanotti, Gardar Sveinbjornsson, Erna V. Ivarsdottir, Hilma Hólm, Daníel F. Guðbjartsson, Ástrós Th. Skúladóttir, Kári Stefánsson, Lincoln Nadauld, Kirk U. Knowlton, Sisse Rye Ostrowski, Erik Sørensen, Ole Birger Vesterager Pedersen, Jonas Ghouse, Søren A. Rand, Henning Bundgaard, Henrik Ullum, Christian Erikstrup, Bitten Aagaard, Mie Topholm Bruun, Mette Christiansen, Henrik K. Jensen, Deanna Alexis Carere, Christopher T. Cummings, Kristen Fishler, Pernille Mathiesen Tørring, Klaus Brusgaard, Trine Maxel Juul, Lotte Saaby, Bo Gregers Winkel, Jens Mogensen, Francesco Fortunato, Giacomo Pietro Comi, Dario Ronchi, J. Peter van Tintelen, Michela Noseda, Michael V. Airola, Imke Christiaans, Arthur A.M. Wilde, Ronald Wilders, Sally-Ann Clur, Arie O. Verkerk, Connie R. Bezzina, Najim Lahrouchi

**Affiliations:** 1 Amsterdam UMC, University of Amsterdam, Heart Center, Department of Clinical and Experimental Cardiology, Amsterdam Cardiovascular Sciences, Amsterdam, the Netherlands; 2 European Reference Network for Rare, Low Prevalence Complex Diseases of the Heart: ERN GUARD-Heart; 3 Department of Human Genetics, Amsterdam UMC, University of Amsterdam, Amsterdam, the Netherlands; 4 Cardiovascular Genetics Center, Montreal Heart Institute and Faculty of Medicine, Université de Montréal, Montreal, QC, Canada; 5 Department of Biochemistry and Cell Biology, Stony Brook University, Stony Brook, NY, USA; 6 deCODE genetics/Amgen, Inc., Reykjavik, Iceland; 7 Faculty of Medicine, University of Iceland, Reykjavik, Iceland; 8 National Heart and Lung Institute, Imperial College London, London, UK; 9 Royal Brompton Hospital, London, UK; 10 British Heart Foundation Centre of Research Excellence, Imperial College London, London, UK; 11 Department of Medical Biology, Amsterdam UMC, University of Amsterdam, Amsterdam, the Netherlands; 12 Cardiovascular Disease Initiative, Broad Institute of MIT and Harvard, Cambridge, MA, USA; 13 University of Groningen, University Medical Centre Groningen, Department of Genetics, Groningen, the Netherlands; 14 Magdi Yacoub Foundation, Cairo, Egypt; 15 NHLI, Imperial College, London, UK; 16 Health in Code, A Coruña, Spain; 17 Medical Department, Dilemma Solutions SL. Cardiovascular Research Group A Coruña University, A Coruña, Spain; 18 Wellcome Sanger Institute, Wellcome Genome Campus, Hinxton, Cambridge, UK; 19 Department of Neurology, Amsterdam UMC, Amsterdam Neuroscience, University of Amsterdam, Amsterdam, the Netherlands; 20 Department of Neuropathology, Amsterdam UMC location University of Amsterdam, Amsterdam Neuroscience, Amsterdam, the Netherlands; 21 Fondazione IRCCS Ca' Granda Ospedale Maggiore Policlinico, Neuromuscular and Rare Disease Unit, Milan, Italy; 22 Intermountain Healthcare, St. George, UT, USA; 23 Intermountain Healthcare, Salt Lake City, UT, USA; 24 Department of Clinical Immunology, Copenhagen University Hospital, Rigshospitalet, Copenhagen, Denmark; 25 Department of Clinical Medicine, Faculty of Health and Medical Sciences, University of Copenhagen, Copenhagen, Denmark; 26 Department of Clinical Immunology, Zealand University Hospital, Køge, Denmark; 27 Department of Cardiology, Copenhagen University Hospital, Rigshospitalet, Copenhagen, Denmark; 28 Statens Serum Institut, Copenhagen, Denmark; 29 Department of Clinical Immunology, Aarhus University Hospital, Aarhus, Denmark; 30 Department of Clinical Medicine, Health, Aarhus University, Aarhus, Denmark; 31 Department of Clinical Immunology, Aalborg University Hospital, Aalborg, Denmark; 32 Clinical Immunology Research Unit, Department of Clinical Immunology, Odense University Hospital, Odense, Denmark; 33 Department of Molecular Medicine, Aarhus University Hospital, Aarhus, Denmark; 34 Department of Cardiology, Aarhus University Hospital, Aarhus, Denmark; 35 GeneDx, LLC, Gaithersburg, Maryland, USA; 36 Department of Pediatrics, Division of Genetics, University of Nebraska Medical Center, Omaha, NE, USA; 37 Munroe-Meyer Institute for Genetics and Rehabilitation, University of Nebraska Medical Center, Omaha, NE, USA; 38 Department of Clinical Genetics, Odense University Hospital, Odense, Denmark; 39 Department of Clinical Genetics, Lillebaelt Hospital, Institute of Regional Health Research, Odense, Denmark; 40 Department of Cardiology, Odense University Hospital, Odense, Denmark; 41 Department of Cardiology, Aalborg University Hospital, Aalborg, Denmark; 42 Dino Ferrari Center, Department of Pathophysiology and Transplantation, University of Milan, Milan, Italy; 43 IRCCS Fondazione Ca' Granda Ospedale Maggiore Policlinico, Neurology Unit, Milan, Italy; 44 Department of Genetics, University Medical Center Utrecht, Utrecht University, Utrecht, the Netherlands; 45 Department of Pediatric Cardiology, Emma Children’s Hospital, Amsterdam UMC, University of Amsterdam, Amsterdam, the Netherlands

**Keywords:** cardiac arrhythmia, sinus node disease, AV conduction defects, population genetics, hypertrophic cardiomyopathy

## Abstract

*POPDC2* encodes the Popeye domain-containing protein 2, which has an important role in cardiac pacemaking and conduction, due in part to its cyclic AMP (cAMP)-dependent binding and regulation of TREK-1 potassium channels. Loss of *Popdc2* in mice results in sinus pauses and bradycardia, and morpholino-mediated knockdown of *popdc2 in* zebrafish results in atrioventricular (AV) block. We identified bi-allelic variants in *POPDC2* in four families with a phenotypic spectrum consisting of sinus node dysfunction, AV conduction defects, and hypertrophic cardiomyopathy. Using homology modeling, we show that the identified variants are predicted to diminish the ability of POPDC2 to bind cAMP. In *in vitro* electrophysiological studies, we demonstrated that, in contrast with wild-type POPDC2, variants found in affected individuals failed to increase TREK-1 current density. While muscle biopsy of an affected individual did not show clear myopathic disease, it showed significantly reduced abundance of both POPDC1 and POPDC2, suggesting that stability and/or membrane trafficking of the POPDC1-POPDC2 complex is impaired by pathogenic variants in either protein. Single-cell RNA sequencing from human hearts demonstrated that co-expression of *POPDC1* and *POPDC2* was most prevalent in AV node, AV node pacemaker, and AV bundle cells. Using population-level genetic data of more than 1 million individuals, we show that none of the familial variants were associated with clinical outcomes in heterozygous state, suggesting that heterozygous family members are unlikely to develop clinical manifestations and therefore might not necessitate clinical follow-up. Our findings provide evidence for bi-allelic variants in *POPDC2* causing a Mendelian autosomal recessive cardiac syndrome.

## Graphical abstract

**Figure:**
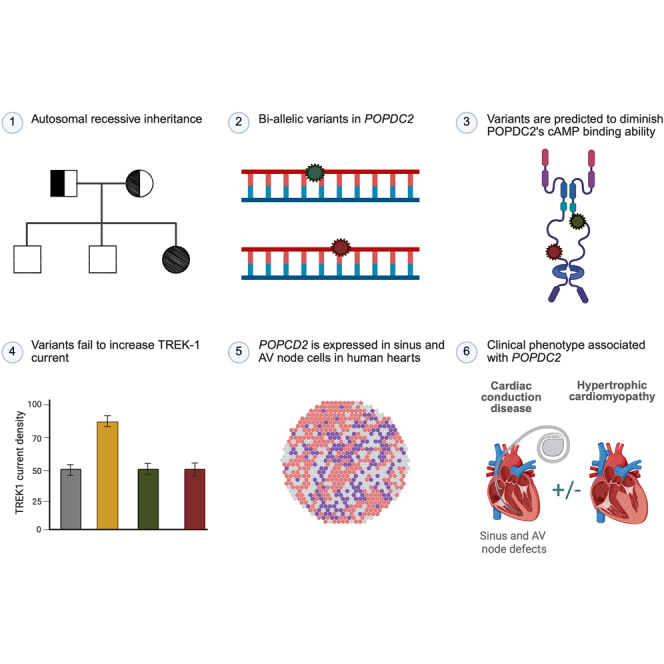

## Teaser

Bi-allelic variants in *POPDC2* cause a recessive syndrome characterized by sinus node dysfunction, AV block, and hypertrophic cardiomyopathy. Modeling and electrophysiological studies suggest effects of identified *POPDC2* variants on cAMP binding and TREK-1 current. Using population-level genetic data, we provide evidence that heterozygous carriers are unlikely to develop clinical disease.

## Introduction

The rhythmic contraction of the heart is orchestrated by the cardiac pacemaker and conduction system.^1^ Electrical activity in the heart arises in the sinus node, located in the right atrium near the entrance of the superior vena cava. The electrical impulse then spreads through the atria to the atrioventricular (AV) node and is subsequently propagated through the bundle of His and bundle branches to the Purkinje fibers from where it spreads throughout the ventricles.

Cardiac conduction defects (CCDs; MIM: 115080) are primarily the consequence of age-related degeneration, structural heart disease, or post-operative complications.^2^ Presentation of CCDs in the young should raise suspicion of a genetic disorder. Rare variants in genes encoding cardiac ion channels (e.g., *SCN5A*, MIM: 600163; *TRPM4*, MIM: 606936; and *HCN4*, MIM: 605206), transcription factors (e.g., *TBX5*, MIM:601620; *NKX2-5*, MIM: 600584), constituents of the inner nuclear membrane (e.g., *LMNA*, MIM: 150330; *EMD*, MIM: 300384), gap junction proteins (e.g., *GJC1*, MIM: 608655), and others (e.g., *GNB5*, MIM: 604447; *TNNI3K*, MIM: 613932; *PRKAG2*, MIM: 602743) have been implicated in inherited CCD presenting in isolation or in presence of other cardiac or extracardiac features.^2^ However, many affected individuals with early-onset CCD remain genetically unexplained.

Bi-allelic variants in *POPDC1* (also known as *BVES*, MIM: 604577), encoding the Popeye domain-containing protein 1, are associated with muscular dystrophy and AV block (MIM: 604577).^3^^,^^4^ In mice, knockout of *Popdc1* or *Popdc2* resulted in stress-induced sinus pauses and sinus bradycardia.^5^ In zebrafish, morpholino knockdown of *popdc1* or *popdc2* resulted in second-degree AV block and bradycardia.^3^^,^^6^ The TWIK-related potassium channel 1 (TREK-1, encoded by *KCNK2*; MIM: 603219) is an established interacting protein of POPDC2^5^ (MIM: 605823) and co-expression of POPDC2 and TREK-1 has been shown to result in a 2-fold higher TREK-1 current in comparison to expression of TREK-1 alone.^5^ Here, we provide evidence for bi-allelic loss-of-function (LOF) variants in *POPDC2* as the cause of an autosomal recessive syndrome in four families, consisting of a phenotypic spectrum including sinus node disease and AV conduction defects with hypertrophic cardiomyopathy (HCM; MIM: 192600).

## Material and methods

### Recruitment and DNA sequencing

Family A was referred to the Department of Human Genetics of the Amsterdam UMC (Amsterdam, the Netherlands) for genetic testing and counseling for CCD and HCM. To follow up on the findings from exome sequencing in this family, we studied 78 individuals that were diagnosed with a similar clinical presentation to family A (i.e., CCDs and HCM, cohorts 1–3). In addition, we studied 96 HCM individuals without CCDs (cohort 4). In all 174 individuals, genetic testing had ruled out causative variants in established arrhythmia and/or cardiomyopathy genes. Families C and D were identified via a genetic and phenotypic match through DECIPHER^7^ and GeneMatcher,^8^ respectively. The study protocol was approved by the Amsterdam University Medical Center Research Ethics Committee and the local Institutional Review Boards of contributing centers. Signed informed consent was obtained from the affected individuals or their parents. Details on case recruitment and DNA-sequencing methods for each family can be found in the supplemental information and Table S1. To ensure the privacy of the affected individuals and their families, (1) ages are presented as non-overlapping age ranges (i.e., 0–5, 6–10, and 11–15 years), (2) pedigrees were modified, (3) information related to ancestry/country or origin/nationality are not reported, and (4) clinical descriptions were minimized. The censoring undertaken for privacy reasons does not affect the ability to evaluate the presented data. Sex was not considered as a biological variable.

### Homology modeling of POPDC2

Two homology models were generated using either SWISS-MODEL^9^ or AlphaFold2 Multimer.^10^ The SWISS-MODEL web server used a cyclic AMP (cAMP)-regulatory protein from *Yersinia pestis* (6DT4) as a template to generate the homology model for the Popeye domain of POPDC2. A dimer of full-length POPDC2 was generated using AlphaFold Multimer,^10^ and the intrinsically disordered C-terminal residues 275–364 were deleted to simplify figure presentation. A final homology model of POPDC2 was created by replacing residues 128–213 of the AlphaFold Multimer model with residues 128–213 of the SWISS-cAMP model after superimposing the individual Popeye domains.

AlphaMissense^11^ was used to generate the predicted pathogenicity of single-amino-acid substitutions and deleted regions. AlphaMissense uses language modeling to understand amino-acid distributions based on sequence context, then it incorporates structural information using an AlphaFold-derived system to consider a protein’s three-dimensional form when assessing a variants’ impact. It also utilizes weak labels from population frequency data to refine predictions without human biases. Using these models, we evaluated the consequence of *POPDC2* variants found in families A–D and five variants that occurred homozygously in individuals from the Genome Aggregation Database v2.1.1 (gnomAD),^12^ which are not expected to cause disease (Table S2)

### *POPDC* expression profiles in single-nucleus and spatial transcriptomics of human hearts

Single-nucleus RNA-sequencing (snRNA-seq) data and Visium Spatial gene expression data were obtained from a previously published study.^13^ Processed data of single-cell RNA-sequencing (scRNA-seq)/snRNA-seq and Visium data are available for browsing gene expression and download from the Heart Cell Atlas (https://www.heartcellatlas.org).^13^ Annotated, log-normalized count matrices for both modalities were downloaded and specifically analyzed for expression of POPDC1, -2, and -3 using Scanpy package for Python run in Jupyter Notebook. Original histological annotation of tissue sections was used. The cell-state annotation was adapted from the original study.^13^ All atrial cardiomyocytes were pooled in one category, and all ventricular cardiomyocytes were pooled together. From the conduction system cells, sinoatrial node pacemaker (SAN P) cells and Purkinje cells are shown separately; due to low cell numbers, atrioventricular node pacemaker (AVN P) and bundle cells are shown together.

### *Popdc* expression profiles in scRNA-seq data in mice

Sinus node and AV node/His scRNA-seq data from mice were obtained from a previously published study.^14^ Using these data, t-distributed stochastic neighbor embedding (t-SNE) maps with a perplexity of 50 were generated on the R2 environment Genomics Analysis and Visualization Platform (http://r2.amc.nl).^15^ The cells were subsequently clustered into different populations using the t-SNE DBSCAN tool. Sentinel gene expression was used to characterize the different clusters (e.g., sinus node cells, expressing higher levels of *Tbx3*, *Isl1*, and *Hcn4*). Thereafter, *Popdc1-3* expression intensities were plotted on the t-SNE maps to identify their expression profiles across the present tissue clusters.

### cDNA constructs and mutagenesis

hTREK-1a cloned in pIRES2-EGFP was obtained from Drs. Delphine Bichet and Florian Lesage (Université Nice Sophia Antipolis, France). Full-length *POPDC2* cDNA sequences (NM_001308333-hg19; wild type [WT], c.516_527del: p.Gln172_Tyr176delinsHis and c.788G>A:p.Arg263His) were synthesized, cloned into pBluescript IISK+ (GeneCust, Boynes, France), and subsequently subcloned into pIRES-GFP (pCGI).

### Electrophysiological characterization of POPDC2 variants

#### Data acquisition

Details on cell preparation and expression can be found in the supplemental information. I_Na_ and TREK-1 currents were measured with ruptured and amphotericin-perforated patch-clamp technique, respectively, using an Axopatch 200B amplifier (Molecular Devices Corporation, Sunnyvale, CA, USA). Voltage control, data acquisition, and analysis were accomplished using custom software. I_Na_ recordings were low-pass filtered with a cutoff frequency of 5 kHz and digitized at 20 kHz, while this was 2 and 4 kHz, respectively, for TREK-1 current measurements. Series resistance was compensated by ≥80%, and potentials were corrected for the calculated liquid junction potential.^16^ Cell membrane capacitance (C_m_) was calculated by dividing the time constant of the decay of the capacitive transient after a −5-mV voltage step from −40 mV by the series resistance. Patch pipettes were pulled from borosilicate glass (Harvard Apparatus, UK) and had resistances of 2.5–3.5 MΩ after filling with the solutions as indicated below. Measurements from a minimum of nine cells from three independent transfections were acquired for each condition.

#### TREK-1 current measurements

TREK-1 currents were recorded at 36 ± 0.2°C. Cells were superfused with solution containing (in mM) NaCl 140, KCl 5.4, CaCl_2_ 1.8, MgCl_2_ 1, glucose 5.5, and HEPES 5 at pH 7.4 (NaOH). Pipette solution contained (in mM) K-gluc 125, KCl 20, NaCl 5, amphotericin-B 0.88, and HEPES 10 at pH 7.2 (KOH). TREK-1 currents were measured using 500-ms voltage-clamp steps to test potentials ranging from −100 to +50 mV from a holding potential of −80 mV. The TREK-1 current was measured at the end of the voltage-clamp step and current densities were calculated by dividing current amplitude by C_m_.

#### I_Na_ measurements

I_Na_ was measured at room temperature using a bath solution containing (in mM) NaCl 20, CsCl 120, CaCl_2_ 1.8, MgCl_2_ 1.0, glucose 5.5, and HEPES 5.0 at pH 7.4 (CsOH). Pipettes were filled with solution containing (in mM) NaF 10, CsCl 10, CsF 110, EGTA 11, CaCl_2_ 1.0, MgCl_2_ 1.0, Na_2_ATP 2.0, and 10 HEPES at pH 7.2 (CsOH). The I_Na_ density and voltage dependence of activation were determined by 50-ms depolarizing pulses to test potentials ranging from −80 to +40 mV from a holding potential of −120 mV. Voltage-dependent inactivation was obtained by measuring the peak currents during a 50-ms test step to −20 mV, which followed a 500-ms prepulse to membrane potentials between −140 and 0 mV to allow inactivation. The holding potential was −120 mV. All voltage-clamp steps were applied with a 5-s cycle length. Peak I_Na_ was defined as the difference between peak and steady-state current. Current density was calculated by dividing the measured currents by C_m_. To determine the activation characteristics of I_Na_, current-voltage curves were corrected for differences in driving force and normalized to maximum peak current (I_max_). Steady-state activation and inactivation curves were fitted using the Boltzmann equation I/I_max_ = A/{1.0 + exp[(V_1/2_ − V)/k]} to determine the membrane potential for half-maximal (in)activation (V_1/2_) and the slope factor k.

#### Statistical analysis

Data are presented as mean ± standard error of the mean (SEM). Statistical analysis was carried out with SigmaStat 3.5 software. Normality and equal variance assumptions were tested with the Kolmogorov-Smirnov and the Levene median test, respectively. Groups were compared with one-way ANOVA. *p* < 0.05 defines statistical significance.

### Computer simulations

The spontaneous electrical activity of a single human sinus nodal pacemaker cell was simulated using the comprehensive mathematical model developed by Fabbri et al.^17^ For simulations of a single human atrial cell, we used the model by Maleckar et al.^18^ The CellML code of both models, as available from the CellML Model Repository^19^ at https://www.cellml.org/, was edited and run in version 0.9.31.1409 of the Windows based Cellular Open Resource (COR) environment.^20^ TREK-1 currents are not included in both original models. To study whether the homozygous loss LOF variants in *POPDC2* contribute to bradycardia via TREK-1 current changes, we fitted our experimental data of the TREK-1 current-voltage relationship (Figure 3A) and implemented the thus-obtained TREK-1 current in both models as a control over a range of TREK-1 current densities. Subsequently, the TREK-1 density was reduced to 59% of the TREK-1 + POPDC2-WT current according to the effects induced by the *POPDC2* variants (Figure 3A) to assess the functional effects of the variants. All simulations were run for a period of 200 s, which appeared a sufficiently long time to reach steady-state behavior. The analyzed data are from the final 10 s of the 200-s period.

### Association of heterozygous *POPDC2* variants and burden analyses for *POPDC2* with clinical phenotypes and heart rate in population biobanks

The recent availability of population-level cohorts with both clinical as well as whole-genome sequencing and well-imputed array genotyping data now provide the opportunity for orthogonal validation of genetic findings through complementary population-level analysis.

#### Biobank description and phenotyping

Samples were included from four large population biobanks (total *n* = 1,089,031) with genetic data, namely deCODE genetics in Iceland (*n* = 173,025), UK Biobank (*n* = 428,503), Copenhagen Hospital Biobank and the Danish Blood Donor study in Denmark (*n* = 487,356), and Intermountain in Utah, USA (*n* = 138,006). Disease status was obtained from electronic health records and ascertained using the following International Classification of Diseases 10th revision codes: atrioventricular block (I44.1 and I44.2), bradycardia (R00.1), cardiac arrest (I46), hypertrophic cardiomyopathy (I42.1 and I42.2), muscular dystrophy (G71.0), myocarditis (I40, I41 and I51.4), and sinus node dysfunction (I49.5). Pacemaker implantation was defined based on procedure codes (deCODE: Nomesco Classification of Surgical Procedures [NCSP] codes FPE/FPSE and FPF/FPSF. Copenhagen Hospital Biobank: NCSP codes FPE/FPSE and FPF/FPSF. UK Biobank: National Clinical Coding Standards OPCS code K60). Heart rate was available only in the UK Biobank and deCODE. In the UK Biobank, heart rate was obtained during blood-pressure measurement at assessment. Both measurements were taken twice, and multiple measurements for one individual were averaged. In deCODE, heart-rate measurements were sourced from electrocardiograms (ECGs) from Landspitali University Hospital in Iceland between 1998 and 2015. Mean values from sinus-rhythm ECGs were obtained for each individual, which were subsequently standardized and adjusted for age and sex. Details on each biobank and DNA genotyping and sequencing methods for each biobank can be found in the supplemental information.

#### Gene burden models

We defined different models to group together various types of variants:

(1) LOF: only LOF variants according to Variant Effect Predictor (VEP).^21^
(2) LOFTEE: high-confidence LOF variants according to LOFTEE.^12^
(3) LOFCADD: LOF and missense (MIS) when predicted deleterious with CADD phred score ≥ 25.^22^
(4) LOF1MISID: LOF and MIS when predicted deleterious by at least one of the following prediction methods: MetaSVM, MetaLR,^23^ or CADD phred score ≥ 25.^22^

In all models, we used minor allele frequency (MAF) <2% to select variants for analyses.

#### Association analyses

For case-control analyses, we used logistic regression under an additive model to test for association between carrying an LOF variant in *POPDC2* (LOF, MIS according to each model A–D) and phenotypes, in which disease status was the dependent variable and genotype counts as the independent variable. For the analyses, we used software developed at deCODE genetics.^24^ For testing association with heart rate, measurements were inverse-normal transformed and analyzed using a linear mixed model implemented in BOLT-LMM.^25^ Meta-analysis was performed on the summary results from IS, UK, DK, and US when available, using a fixed-effects inverse-variance-weighted method.^26^

## Results

### *POPDC2* variants cause sinus node disease and AV conduction defects with HCM in multiple families

**Figure 1:**
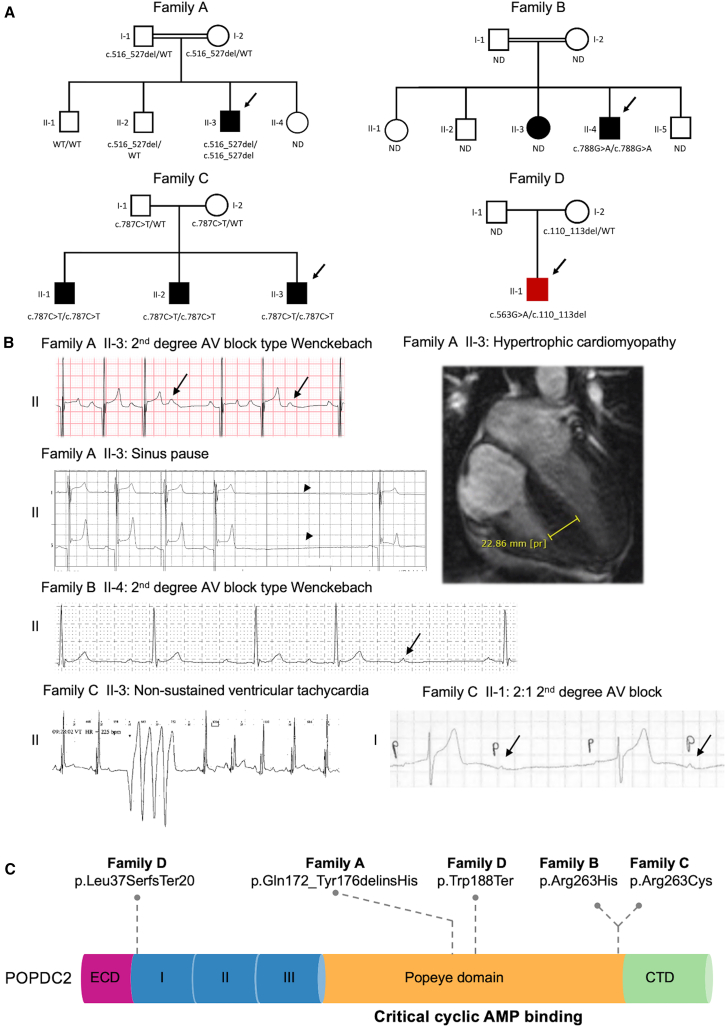
Bi-allelic variants in *POPDC2* cause a recessive syndrome with sinus node disease and atrioventricular conduction defects with HCM (A) Pedigrees of families A–D. Closed symbols indicate affected individuals. Males are indicated by squares and females by circles. A double line indicates a consanguineous relationship. The arrows point to the probands. Affected individual 6 from family D (highlighted in the pedigree with a filled red box) was diagnosed with bradycardia resulting in an arrest and first-degree AV block during an episode of fulminant myocarditis. (B) Selection of ECG abnormalities: (1) affected individual II-3 from family A (second-degree AV block type Wenckebach and sinus pause [indicated by an arrowhead]; see Figure S1 for longer Holter registration), (2) affected individual II-4 from family B (second-degree AV block type Wenckebach), affected individual II-3 (non-sustained ventricular tachycardia), and affected individual II-1 (2:1 second-degree AV block) from family C. Arrows point to a non-conducted P wave. Upper right panel: cardiac MRI at the age of 11–15 years showing marked hypertrophy of the interventricular septum (23 mm, *Z* score^27^: 16.43; height, 160 cm; weight, 49 kg) in the proband of family A. (C) POPDC2 protein domain structure and location of variants found in affected individuals. AV, atrioventricular; CTD, carboxy-terminal domain; ECD, extracellular domain; ND, genotype not determined; regions I/II/II, transmembrane region 1–3; WT, wild type.

To uncover the genetic cause of sinus node disease, AV conduction defects and HCM in a child (age at presentation 11–15 years; family A), we performed whole-exome sequencing in the proband, both parents, and two of his unaffected siblings. His parents are first cousins (Figure 1, individual II-3 in the pedigree; Tables 1 and S3; Figure S1).

**Table 1:** Clinical characteristics of affected individuals with bi-allelic *POPDC2* variants

	**Family A**	**Family B**	**Family C**			**Family D**
	**II-3**	**II-4**	**II-1**	**II:2**	**II:3**	**II-1**
POPDC2 variant	p.Gln172_Tyr176delinsHis	p.Arg263His	p.Arg263Cys			p.Trp188Ter; p.Leu37Serfs^∗^20
Effect on TREK1 current	LOF	LOF	not tested			not tested
Consanguinity	yes	yes	no			no
Sex	M	M	M	M	M	M
Age at presentation (years)	11–15	21–25	21–25	21–25	16–20	11–15
Symptoms at presentation	palpitations	palpitations	none	none	cardiac arrest	chest pain

**Cardiac arrhythmia and ECG abnormalities**						

Cardiac arrest	no		no	no	arrest following bradycardia	arrest following bradycardia
Minimum heart rate (BPM)	33	30	33	N/A	N/A	67
Sinus node disease	sinus pauses (3 s)	sinus bradycardia	no	SA-block sinus pauses (5 s)	SA block sinus arrest	no
AV conduction disease	2^ND^ AVB (type 1)	PQ time of 200 ms second AVB	first and second AVB (type 1 and 2:1)	first AVB	first and second AVB (type 1)	first AVB
Atrial arrhythmia	atrial fibrillation and flutter	no	atrial fibrillation and flutter	no	episodes of high atrial rate	no
Ventricular arrhythmia	monomorphic NSVT, PVCs	no	monomorphic NSVT	no	monomorphic NSVT	VT

**Structural cardiac and extracardiac abnormalities**						

Hypertrophic cardiomyopathy	yes	yes	no	no	no	no
Cardiac MRI findings (mm)	septal hypertrophy (23 mm)	septal hypertrophy (16mm)	N/A	N/A	possible myocarditis	significant LV fibrosis and inflammation
Myocarditis	no	no	no	no	possible myocarditis	clinically (not on cardiac histology)

**Treatment**						

Pacemaker implantation (age, years)	yes (15)	no	yes (23)	yes (21)	yes (17)	yes (15), temporary
ICD implantation (age, years)	yes (15)	no	no	no	yes (33)	no
Appropriate ICD shocks	no	N/A	N/A	N/A	no	N/A

AVB, atrioventricular block, BPM, beats per minute; ICD, implantable cardiac defibrillator; MRI, magnetic resonance imaging; N/A, not available, NSVT, non-sustained ventricular tachycardia; PVC, premature ventricular contractions; SA, sinoatrial; TTE, transthoracic echocardiogram; VT, ventricular tachycardia.

No likely causal variant was found in genes previously associated with Mendelian cardiomyopathies or arrhythmia syndromes (either recessive or dominant; Table S4). In line with the expected recessive inheritance pattern, we identified a rare segregating homozygous variant, NM_001308333.2:c.516_527del; NP_001295262.1:p.(Gln172_Tyr176delinsHis), in the Popeye domain-containing protein 2 (*POPDC2*), as the most likely variant consistent with a recessive mode of inheritance and parental consanguinity (see Table S5 for overview of the two segregating coding-region variants identified in the homozygous state). Of note, the only other homozygous segregating variant has been found 15 times in gnomAD and was therefore found unlikely to cause the disease in this individual. No *de novo* variants were found in the index affected individual. The cardiac arrhythmia phenotype in the affected individual is consistent with studies in mice that showed that loss of *Popdc2* resulted in sinus pauses and bradycardia^5^ and with findings made in zebrafish where morpholino knockdown of *popdc2* resulted in AV block.^6^ We therefore found the p.Gln172_Tyr176delinsHis variant as an excellent candidate for this cardiac disorder. We then searched for additional individuals carrying bi-allelic variants in *POPDC2* by screening this gene in 78 individuals that presented with a similar phenotype to family A (i.e., CCD with HCM) and a search using GeneMatcher^8^ and DECIPHER.^7^ In total, we identified six affected individuals from four unrelated families of different ancestries (affected individuals 3–5 are siblings, Figures 1A and 1B; Tables 1 and S3) who harbor either homozygous or compound heterozygous rare *POPDC2* variants. All six affected individuals were diagnosed with CCDs with or without HCM.

In summary (see Tables 1 and S3; Figure 1B), (1) AV conduction disease was present in all six individuals, mainly consisting of first-degree AV block or second-degree AV block type 1 (Wenkebach), (2) sinus node disease presenting as sinus bradycardia and sinus pauses was present in four out of six individuals, (3) cardiac arrest accompanied by sinus bradycardia or asystole was seen in two out of six individuals, (4) atrial arrhythmia (i.e., atrial flutter or fibrillation) was detected in three out of six individuals, (5) non-sustained ventricular tachycardia occurred in four out of six individuals, and (6) HCM was diagnosed in two out of six individuals (probands in families A and B with no other genetic variant causing HCM). A pacemaker was implanted in five out of six affected individuals with the age at implantation ranging from 15 to 23 years. In two out of six affected individuals, an implantable cardioverter-defibrillator (ICD) was implanted to prevent lethal ventricular arrhythmia (ranging from 15 to 33 years), but no appropriate ICD shocks occurred. Unlike the recessive syndrome associated with the other POPDC genes (i.e., *POPDC1* and *POPDC3*-related limb-girdle muscular dystrophy), none of the affected individuals showed signs of muscular dystrophy. Of note, individual 6 (II-1 in family D; Figure 1A) presented with acute myocarditis, for which he was admitted to the intensive care unit. Detailed phenotypic descriptions of all affected individuals can be found in Note S1.

We noted an autosomal recessive mode of inheritance in families A and C wherein the identified variants in *POPDC2* were inherited from each of the unaffected parents. While we expect a recessive inheritance, we could not assess the inheritance pattern in individuals 2 and 6 (from family B, II-4; and D, II-1; Figure 1A) as DNA of (one of) the parents was not available. In total, we report five *POPDC2* variants (RefSeq transcript: NM_001308333.2; protein ID: NP_001295262.1), of which two are missense variants (c.788G>A [p.Arg263His], c.787C>T [p.Arg263Cys]), one in-frame insertion deletion (c.516_527del [p.Gln172_Tyr176delinsHis]) and two are expected to result in protein truncation (c.563G>A [p.Trp188Ter], c.110_113del [p.Leu37SerfsTer20], Table S2). The MAF of *POPDC2* variants identified in affected individuals ranged from 1.4 × 10^−5^ to 1.3 × 10^−4^ in the gnomAD v4.0 (accessed March 2024; Table S2). Furthermore, none of the variants were found homozygous in 730,947 exomes and 76,215 genomes from gnomAD, suggesting that homozygosity for these variants is not well tolerated. In line with this, all variants are predicted damaging by multiple *in silico* prediction tools (Table S2). Intriguingly, in families B and C, the same residue is affected: p.Arg263His and p.Arg263Cys, respectively. Of note, a homozygous variant (c.787G>A [p.Arg261Gln]) in *POPDC3*, which affects the paralogous residue of p.Arg263 in *POPDC2* (families B and C), has been associated with muscular dystrophy without cardiac arrhythmia (MIM: 604577).^28^ In family C, the three affected siblings homozygous for p.Arg263Cys inherited the variant from the heterozygous and clinically unaffected parents (II-1, II-2, and II-3 in Figure 1A). We performed a look-up of the p.Arg263Cys variant in recent whole-genome sequencing dataset^29^ consisting of young affected individuals (*n* = 226) receiving a pacemaker because of AV block, and none of the individuals was homozygous for p.Arg263Cys, nor were there any individuals with bi-allelic variants in *POPDC2* (Note S2). In family D, we identified both p.Trp188Ter and p.Leu37Serfs^∗^20 variants that are expected to result in LOF due to premature truncation of the protein and/or nonsense-mediated decay (individual II-1 in Figure 1A). The cardiac arrest accompanied by sinus bradycardia and first-degree AV block he displayed fitted the conduction disease seen in the other *POPDC2*-affected individuals. However, although the cardiac biopsy was negative for myocarditis, a potential causal role of myocarditis cannot be ruled out, as the disease can be focal and patchy, potentially reducing sensitivity due to sampling error.

### Homology model for POPDC2

To explore the structural and functional consequences of the homozygous and compound heterozygous *POPDC2* variants, we generated a predicted structural model of POPDC2, as no experimentally determined structures are currently available. SWISS-MODEL^9^ and AlphaFold Multimer^10^ were used since these protein-structure-prediction programs could generate dimeric models of POPDC2, and dimerization has been shown to be critical for the activity of POPDC1.^25^ As a complementary approach, AlphaMissense^11^ was used to predict relative pathogenicity scores of the identified single-amino-acid substitutions and deleted regions.

**Figure 2:**
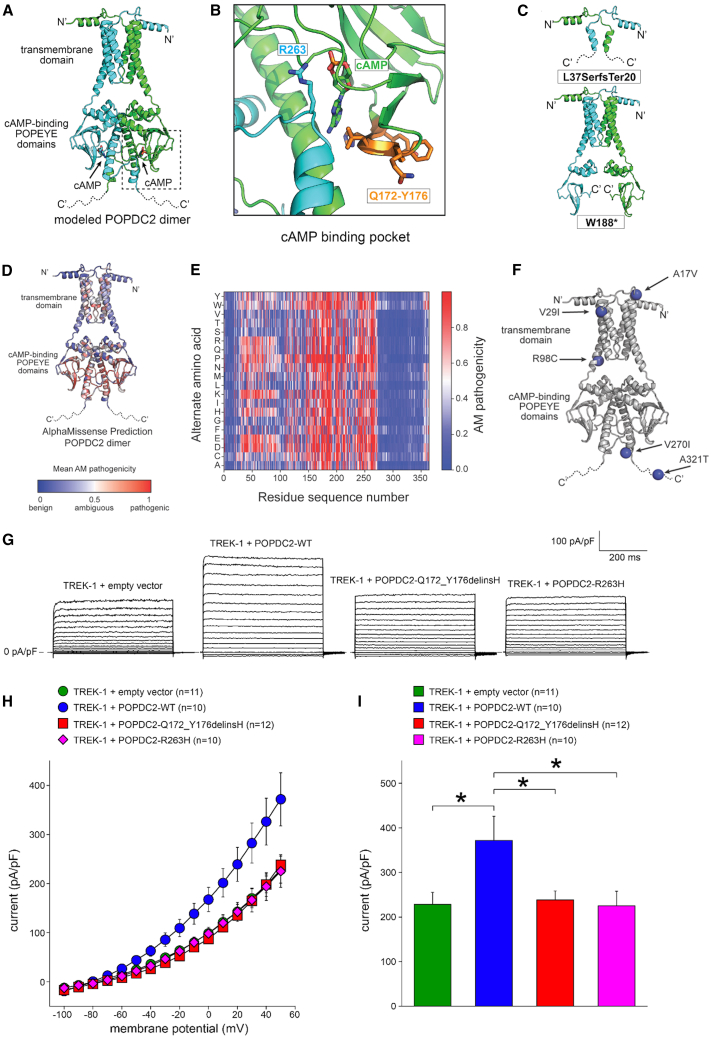
Functional characterization of the *POPDC2* variants (A) Structural model of POPDC2 bound to cAMP generated using AlphaFold2 Multimer and SWISS-MODEL. Dimer subunits are shown in green and cyan; cAMP molecules, green and cyan sticks. N′ and C′ indicate the N and C termini. The positions of the transmembrane and Popeye domains are indicated by the labels. The intrinsically disordered C termini (residues 275–364) are shown as dotted lines. (B) Zoom-in of the predicted cAMP-binding pocket of POPDC2. Dimer subunits are shown in green and cyan; cAMP, green sticks; residues p.Gln172_Tyr176delinsHis, orange; p.Arg263, cyan sticks. (C) Structural models of the POPDC2 variants p.Leu37Serfs^∗^20 and p.Trp188Ter, which would both generate truncated proteins that would lack the ability to bind cAMP. (D) Homology model of POPDC2 by AlphaFold Multimer color coded by the average pathogenicity score for each residue as predicted by AlphaMissense. (E) Heatmap of predicted effects of amino-acid substitutions on POPDC2. AlphaMissense (AM) scores range from zero to one, with higher scores corresponding to increased pathogenicity. (F) Homology model of POPDC2 by AlphaFold Multimer color coded by the average pathogenicity score for each residue as predicted by AlphaMissense. The position of non-disease-associated variants found in general population are shown as blue spheres, indicating predicted AlphaMissense pathogenicity scores <0.1. (G) Typical examples of TREK-1 currents upon 500-ms voltage-clamp steps to membrane potentials ranging from −100 to +50 mV from a holding potential of −80 mV in absence or presence of wild-type (WT) and mutant POPDC2. (H) Average current-voltage relationships of TREK-1 currents in absence or presence of WT and mutant POPDC2. (I) TREK-1 current amplitude at +50 mV in absence or presence of WT and mutant POPDC2. ^∗^*p* < 0.05 with one-way ANOVA. Error bars indicate the standard error of the mean (SEM).

AlphaFold Multimer generated a high-confidence dimer (Figure S2A) for full-length POPDC2 with a dimeric transmembrane domain composed of six transmembrane α helices (three α helices from each subunit) and two cAMP-binding Popeye domains that also contained an extensive dimer interface (Figure 2A). In the AlphaFold model, Arg263 (altered in families B and C) was pointing into the cAMP-binding pocket of the Popeye domain from the adjacent subunit. SWISS-MODEL was the only program able to model a cAMP-bound state of the Popeye domain (Figure S2B). To generate a model of the cAMP-bound state of full-length POPDC2, we merged the AlphaFold Multimer and SWISS-MODEL predictions by swapping the Popeye domains (see section material and methods). This created a merged model of POPDC2 that included the transmembrane domain and the Popeye domains bound to cAMP (Figure 2A).

Based on the structural model, all variants (p.Gln172_Tyr176delinsHis, p.Arg263His, p.Arg263Cys, and p.Trp188Ter/p.Leu37Serfs^∗^20) are predicted to diminish the ability of POPDC2 to bind cAMP. The first variant (p.Gln172_Tyr176delinsHis, family A) replaces five residues with a single histidine residue. The deleted residues are predicted to form one beta strand that is part of a three-stranded beta sheet at the base of the cAMP-binding pocket (Figure 2B). Replacement of the five residues with a single histidine residue would significantly alter the structural integrity of the cAMP-binding Popeye domain and directly affect the ability of POPDC2 to bind cAMP.^25^ The second and third variants, p.Arg263His (found in family B) and p.Arg263Cys (found in family C), are also both predicted to interfere with proper cAMP binding, as the positively charged Arg residue is predicted to be near the negatively charged cyclic phosphate group of cAMP (Figure 2B). Change of the Arg to either His or Cys would eliminate a key interaction predicted to stabilize cAMP binding and thus reduce or eliminate the ability of POPDC2 to bind cAMP. In support of Arg263 being involved in a specific interaction to stabilize cAMP binding, alteration of Arg263 to any other residue is predicted to be pathogenic by AlphaMissense, with pathogenicity scores of 0.82 and 0.79 for the p.Arg263His and p.Arg263Cys variants, respectively.

The compound heterozygous variants are also predicted to eliminate cAMP binding by POPDC2 (Figure 2C). p.Leu37SerfsTer20 is a truncated protein that completely lacks the cAMP-binding Popeye domain and would also generate an incomplete transmembrane domain. p.Trp188Ter would retain the dimeric transmembrane domain, but truncate the Popeye domain, thus leaving an incomplete domain incapable of binding to cAMP (Figure 2C). In addition, both variants could be subject to nonsense-mediated decay, which would result in no protein product being made.

Notably, residues with high pathogenicity scores (>0.5) all cluster in the regions of the POPDC2 structure that are involved in either dimerization or cAMP binding (Figures 2D and 2E), which emphasizes the importance of cAMP binding and dimerization for POPDC2 function. Consistently, other variants within POPDC2, found homozygously in gnomAD,^12^ and without a clear disease association (p.Arg17Val, p.Val29Ile, p.Arg98Cys, p.Val270Ile, and p.Ala321Thr), are all positioned away from the cAMP-binding pocket and are predicted to be benign by AlphaMissense with low pathogenicity scores <0.1 (Figures 2E and 2F). Taken together, the disease-associated variants reported here are primarily located in regions of the POPDC2 structure that are critical for protein function.

### POPDC2 variants fail to increase TREK-1 current density

We then aimed to functionally characterize the POPDC2 variants. TREK-1 is a recognized interacting protein of POPDC2,^5^ and co-expression of POPDC2 and TREK-1 has been shown to increase TREK-1 current in comparison to expression of TREK-1 alone.^5^ Furthermore, cardiac-specific TREK-1-deficient mice display a sinus node phenotype characterized by bradycardia with frequent episodes of sinus pauses, partially resembling the phenotype in the affected individuals with bi-allelic *POPDC2* variants presented here.^30^ We therefore hypothesized that the effect of the p.Gln172_Tyr176delinsHis (family A) and p.Arg263His (family B) variants is mediated through modulation of the TREK-1 current. We did not test the variants found in family C (p.Arg263Cys) and family D (p.Trp188Ter/p.Leu37Serfs^∗^20) as they affect the same residue as the variant in family B (p.Arg263His) or are expected to result in premature truncation of the protein and possibly nonsense-mediated decay, respectively. We co-transfected HEK293 cells with WT and mutant POPDC2 with TREK-1 containing plasmids. As expected,^5^ co-expression of WT POPDC2 with TREK-1 increased TREK-1 current density (Figures 2G–2I). However, when we co-expressed TREK-1 with a p.Gln172_Tyr176delinsHis or p.Arg263His POPDC2-containing plasmid, no increase in TREK-1 current density was observed, comparable to the co-expression of TREK-1 with an empty vector (Figures 2G–2I). We then tested the effect of the POPDC2 variants on sodium current (I_Na_) as it was recently hypothesized that POPDC2 may interact with Na_V_1.5.^31^ However, expression of WT, p.Gln172_Tyr176delinsHis, or p.Arg263His POPDC2 in an HEK293 cell line stably expressing human Na_V_1.5 channels (encoded by *SCN5A*) showed an effect neither on I_Na_ density nor on I_Na_ gating properties by WT or mutant POPDC2 (Figure S3). Western blot analysis showed levels of expression of the mutant proteins that were comparable to the levels seen for WT POPDC2 (Figure S4).

### *In silico* modeling of the decrease in TREK-1 current induced by the POPDC2 variants on human sinus node and atrial cells

**Figure 3:**
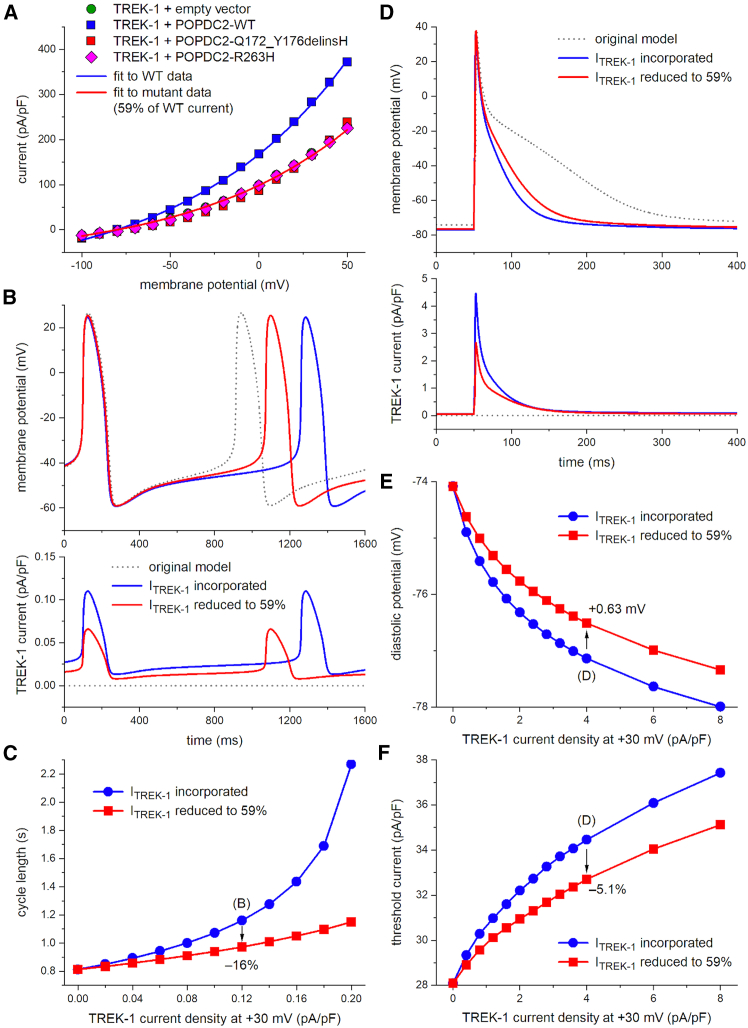
Functional effects of the *POPDC2* variants from *in silico* modeling (A) Fits to the experimental data on TREK-1 currents in absence or presence of wild-type (WT) and mutant POPDC2. The fit to the mutant data were obtained by scaling the fit to the WT data by a factor of 0.59. (B) Membrane potential (top) and associated TREK-1 current (bottom) of a single human sinus nodal pacemaker cell as simulated using the comprehensive mathematical model developed by Fabbri et al.^17^ I_TREK-1_ was introduced into the original model cell using the fits of (A). I_TREK-1_ magnitude was set to 0.12 pA/pF at a membrane potential of +30 mV (C) Cycle length of the simulated single human sinus nodal pacemaker cell as a function of I_TREK-1_ magnitude. (D) Membrane potential (top) and associated TREK-1 current (bottom) of a single human atrial cell as simulated using the comprehensive mathematical model developed by Maleckar et al.^18^ I_TREK-1_ was introduced into the original model cell using the fits of (A). I_TREK-1_ magnitude was set to 4.0 pA/pF at a membrane potential of +30 mV. Action potentials were elicited at a rate of 1 Hz with a 1-ms, 20% suprathreshold stimulus current. (E) Diastolic potential of the simulated single human atrial cell as a function of I_TREK-1_ magnitude. (F) Threshold stimulus current of the simulated single human atrial cell as a function of I_TREK-1_ magnitude.

To evaluate whether the observed decrease in TREK-1 current density, associated with the POPDC2 variants, is responsible for the bradycardia observed in affected individuals, we conducted computer simulations using comprehensive mathematical models of both a human sinus nodal pacemaker cell^17^ and a human atrial cell.^18^ As shown in Figure 3A (blue line), the experimental data on the voltage dependence of the TREK-1 + POPDC2-WT current could be well fitted (r^2^ > 0.99) with the relationship I_TREK-1_ = −117.1 + 284.24 × exp(V_m_/90.52), in which I_TREK-1_ and V_m_ denote TREK-1 current density (in pA/pF) and membrane potential (in mV), respectively. The mutant data could be well fitted by scaling down I_TREK-1_ to 59% of the TREK-1 + POPDC2-WT current over the entire volage range (Figure 3A, red line). We started our simulations with incorporating I_TREK-1_, which is not present in the original model cell, into the human sinus node model cell. Because data on the density of I_TREK-1_ in human sinus node cells are lacking, we set its density to 1.2 pA/pF (at +30 mV), as observed in mouse sinus node cells by Unudurthi et al.^30^ This, however, resulted in cessation of pacemaker activity in the human sinus node model cell. We then repeated our simulations with a 10 times lower density of I_TREK-1_. With this density, the cycle length of the simulated action potential amounted to 1,160 ms (Figure 3B, top panel, blue solid line), as compared to 813 ms in the original model (Figure 3B, top panel, gray dotted line). Cycle length was reduced by 16% from 1,160 to 973 ms upon reduction of I_TREK-1_ density to 59%, thus simulating the effect of the variants in *POPDC2* (Figure 3B, top panel, red solid line; Figure 3C, vertical arrow). The decrease in cycle length was mainly due to an increase in diastolic depolarization rate (Figure 3B, top panel) as a result of the decrease in the small but effective I_TREK-1_ during this phase of the action potential (Figure 3B, bottom panel). Qualitatively similar results were obtained with other I_TREK-1_ densities. The observed effects on cycle length are summarized in Figure 3C.

One may argue that the POPDC2 variant-induced decrease in I_TREK-1_ affects pacemaker activity by changing the excitability of the atrial tissue surrounding the sinus node. This was assessed in simulations of a human atrial cardiomyocyte after incorporating I_TREK-1_, which was not represented in the original model cell.^18^ Figure 3D shows the results obtained with an I_TREK-1_ density of 4.0 pA/pF (at +30 mV). Simulating the effect of the variants in *POPDC2* by lowering the density of I_TREK-1_ to 59% led to a 0.63-mV depolarization of diastolic potential, a 5.1% decrease in threshold current (Figures 3E and 3F, vertical arrows), and a 23-ms increase in action-potential duration (APD) at 90% repolarization. The relatively small effects on diastolic potential and threshold current were obtained with a probably overestimated I_TREK-1_ density, given the large effect of I_TREK-1_ on the APD of the original model (Figure 3D, top panel). Smaller, but qualitatively similar, effects on diastolic potential and threshold current were obtained with lower I_TREK-1_ densities (Figures 3E and 3F), associated with a less prominent effect of I_TREK-1_ on the APD of the original model. Overall, despite the observation of bradycardia in mice lacking TREK-1, findings from our simulation studies do not provide an explanation for the clinically observed sinus bradycardia in individuals with *POPDC2* variants based on a decrease in I_TREK-1_ per se.

### p.Gln172_Tyr176delinsHis results in a significant reduction of both POPDC1 and POPDC2 abundance in skeletal muscle

**Figure 4:**
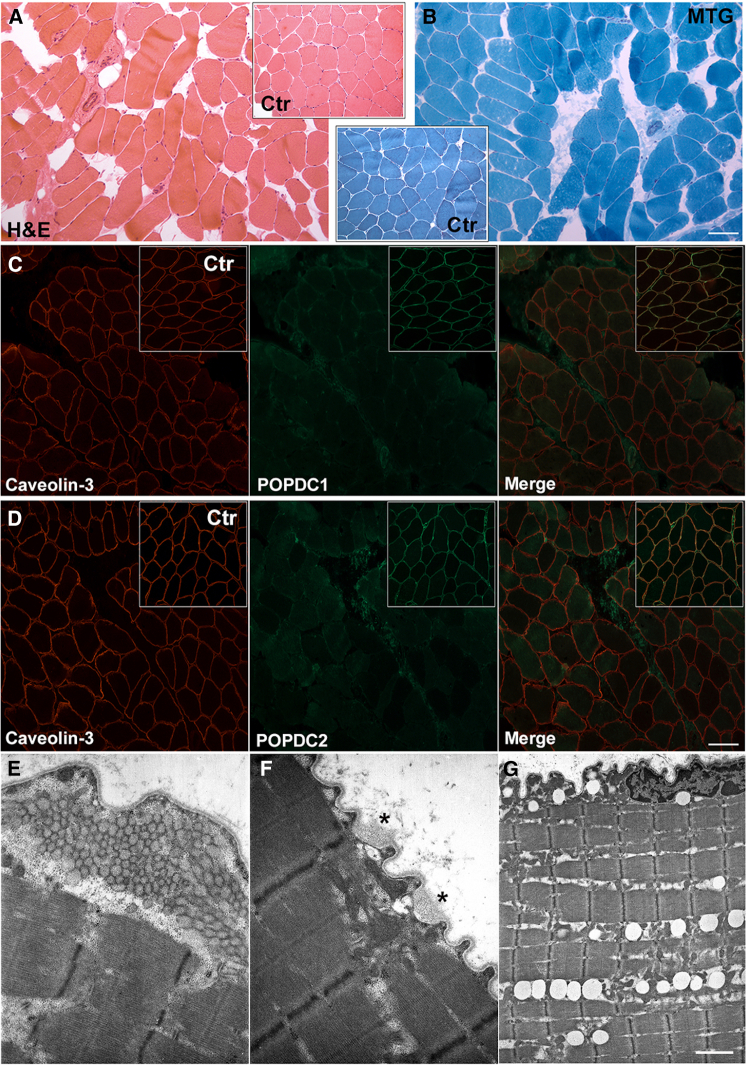
Evaluation of muscle biopsy from the proband in family A (A and B) (A) Hematoxylin and eosin (H&E) stain and (B) modified Gomori trichrome (MGT) staining of affected individual and, in the inset, control muscle. Scale bar, 50 μm. (C) Immunofluorescent staining for caveolin-3 (red), POPDC1 (green), and merge for affected individual. The inset shows the corresponding immunofluorescence staining for control. Scale bar, 50 μm. (D) Immunofluorescent staining for caveolin-3 (red), POPDC2 (green), and merge for affected individual. The corresponding control staining is shown in the inset. Scale bar, 50 μm. (E–G) Ultrastructural findings. (E) Tubular aggregates in subsarcolemmal region. (F) Sarcolemma alteration (asterisks). (G) Increase in lipid droplets. Scale bar (E and F), 0.84 μm; (G), 3.33 μm.

Individual II-3 from family A (Figure 1A) underwent muscle biopsy of the m. vastus lateralis (Supplemental information). Histological analysis disclosed mild fiber size variability and a slight increase of connective tissue. Neither nuclear centralizations nor fiber splittings were observed. No necrotic or degenerated fibers were detected (Figures 4A and 4B). Non-specific myopathic features and increased connective tissue were previously observed in muscle samples from individuals with pathogenic *POPDC1* variants,^3^^,^^32^^,^^33^ while affected individuals with *POPDC3* variants displayed typical features of muscle dystrophies, although the severity of the histopathological findings differed among affected individuals.^28^

In healthy skeletal muscle fibers, POPDC1 and POPDC2 were robustly localized in the sarcolemma. Conversely, in the affected individuals’ muscle, immunofluorescence staining showed a significant reduction of POPDC1 (Figure 4C) and POPDC2 (Figure 4D) levels. On the other hand, the immunofluorescent signal for caveolin-3, which also stains muscle membranes, showed normal distribution and intensity in the muscle sample of the affected individual II-3 from family A (Figure 1A) compared to control. The severe reduction of POPDC1 and POPDC2 levels was also documented by SDS-PAGE analysis of muscle protein lysates (Figure S5). These findings suggest that POPDC2 p.Gln172_Tyr176delinsHis affects the stability of POPDC2, hampering its membrane localization and leading to the secondary reduction of POPDC1. Indeed, the stability and/or membrane trafficking of the POPDC1-POPDC2 complex have been found to be impaired by genetic variants in each of the two proteins.^3^^,^^33^

Ultrastructural examination detected the presence of tubular aggregates in few muscle fibers (Figure 4E). Alterations in the structure of the sarcolemma, characterized by small microvilli-like projections, together with subsarcolemmal vacuoles were also observed. Basal lamina appeared unstructured and enlarged in some fibers (Figure 4F, asterisks). We also noted increased level of lipids, which in some cases were arranged to form rows of droplets (Figure 4G). Heterogeneous transmission electron microscopy findings were previously observed in muscle samples from affected individuals harboring variants in *POPDC1*^3^^,^^32^ but not in those with *POPDC3* pathogenic variants (MIM: 605824).^28^

### *POPDC2* expression in human hearts

**Figure 5:**
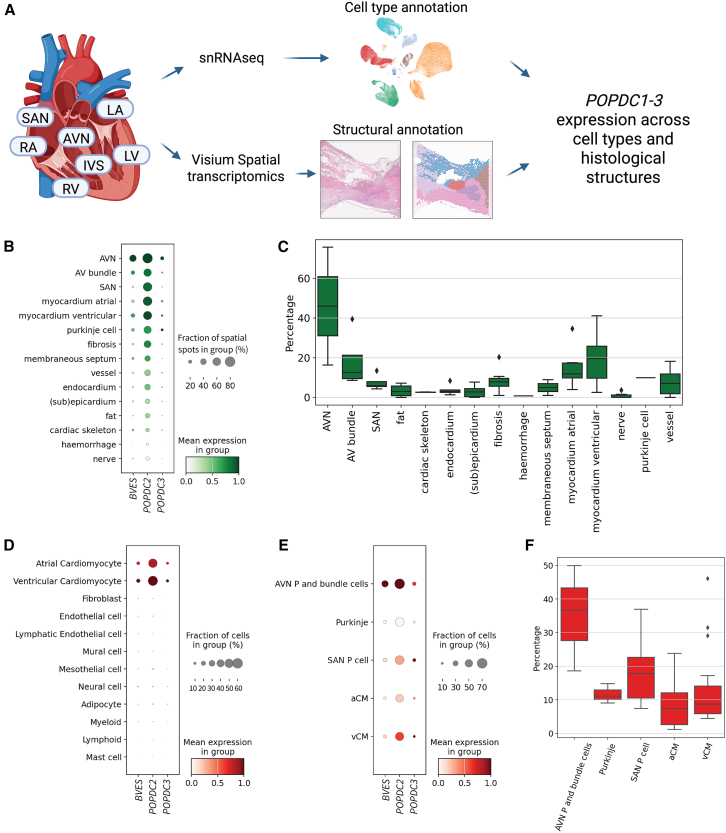
Expression of *POPDC1-3* in human hearts (A) Overview of single-nucleus and spatial-transcriptomics data analysis from a previously published human heart cell atlas.^13^. (B) Spatial-transcriptomics (Visium) analysis of *POPDC1* (*BVES*), *POPDC2*, and *POPDC3* expression across different anatomical regions and histological microstructures in adult human hearts. The anatomical sites sampled included AVN, SAN, left ventricle free wall, left ventricular apex, interventricular septum, left atrium, and right atrium. (C) Percentage of spatial spots where co-expression of both POPDC1/BVES and -2 was detected is shown for each histological feature. (D) *POPDC* family gene expression across cell types in adult human heart profiled by snRNA-seq (10× Genomics). (E) *POPDC1/BVES*, -*2*, and -*3* gene expression in cardiomyocyte cell states in adult human hearts. (F) Percentage of cells that co-express *POPDC1/BVES* and *POPDC2* in the same single cardiomyocyte. Figure created with BioRender. Error bars indicate the 95% confidence interval.

To explore the anatomical distribution of *POPDC1-3* expression (POPDC1’s previous official gene name is BVES), we analyzed a previously published Visium Spatial and single-nucleus transcriptomic dataset.^13^ For the Visium Spatial dataset, annotation of histological tissue sections of the AV node and the sinus node was used and expression of *POPDC1-3* was explored across those structures (Figure 5A). *POPDC2* showed higher expression than *POPDC1/BVES* and *POPDC3*. Cardiomyocyte-rich structures showed higher *POPDC2* expression compared to cardiomyocyte-poor regions (e.g., cardiac skeleton or fat) (Figure 5B). We then interrogated the co-expression pattern of *POPDC1/BVES* and *POPDC2*, as (1) Swan et al. described the necessity of *POPDC1-2* co-expression for their trafficking to the cell membrane, which is required for their proper function^33^; and (2) variants in *POPDC1/BVES-POPDC2* have also been found to affect the stability and/or membrane trafficking of the complex^3^^,^^33^ as corroborated here in the muscle biopsy of the affected individual II-3 from family A (Figure 4). The most prominent area where *POPDC1/BVES* and -*2* expression was seen co-localizing in the same Visium spots was the AV node (Figure 5C). The sinus node expressed *POPDC2*, but expression of *POPDC1* was sparse (Figure 5B).

To validate these findings and to explore *POPDC1* and -*2* co-expression within the same cell, we analyzed snRNA-seq data from eight regions of adult human hearts, including the conduction system. *POPDC2* expression was almost entirely restricted to atrial and ventricular cardiomyocytes compared to other cell types (Figure 5D).^13^ Other genes from the *POPDC* family (i.e., *POPDC1* and *POPDC3*) also showed a preferential expression in cardiomyocytes, but their expression was less prevalent than *POPDC2*. The snRNA-seq object was then sub-set to cardiomyocytes only and expression of *POPDC1-3* interrogated. *POPDC2* was expressed in all cardiomyocyte cell states, while *POPDC1* showed the highest expression in the pacemaker cells (P cells) of the AV node and the AV bundle cells (Figure 5E). The co-expression of *POPDC1* and -*2* within the same single-cell was most prevalent in AV node P cells and AV bundle cells, which concurs with the spatial transcriptomic data (Figure 5F). We then also assessed the cardiac expression of *Popdc2* in mice utilizing previously published^14^ scRNA-seq data of the sinus node and AV node/His region, dissected from embryonic-day-16.5 mouse hearts. Consistent with the phenotype observed in the affected individual, *Popdc2* was expressed in cardiomyocytes as well as sinus node and AV node/His-region cells (Figure S6), corroborating observations from previous studies in mice^5^^,^^34^ and our observations reported here in human hearts (Figures 5A–5F).

### Heterozygous carriers of rare *POPDC2* variants do not display characteristics of the recessive *POPDC2* syndrome at the population level

We investigated whether heterozygous carriers of the *POPDC2* variants found in families A–D (i.e., p.Gln172_Tyr176delinsHis, p.Arg263His, p.Arg263Cys, p.Trp188Ter, and p.Leu37Serfs^∗^20) showed (sub-)clinical manifestations of the recessive *POPDC2* syndrome in four large population biobanks (total *n* = 1,089,031) with genetic data, namely deCODE genetics in Iceland (*n* = 173,025), UK Biobank (*n* = 428,503), Copenhagen Hospital Biobank and the Danish Blood Donor study in Denmark (*n* = 487,356), and Intermountain in Utah, USA (*n* = 138,006). We searched their association with (1) AV block, (2) bradycardia, (3) cardiac arrest, (4) HCM, (5) myocarditis, (6) pacemaker implantation, (7) sinus node dysfunction, and (8) heart rate. We detected 426 heterozygous carriers of the five familial *POPDC2* variants (p.Gln172_Tyr176delinsHis, *n* = 62; p.Arg263His, *n* = 34; p.Arg263Cys, *n* = 156; p.Trp188Ter, *n* = 162; and p.Leu37Serfs^∗^20, *n* = 12) among 1,089,031 participants in the included biobanks but no homozygotes or compound heterozygotes. None of the variants showed statistical association with (sub-)clinical outcomes after Bonferroni correction, suggesting that heterozygous family members are unlikely to develop clinical manifestations and therefore might not necessitate clinical follow-up (Table S6). We then conducted genetic burden analyses, wherein rare variants in *POPDC2* are aggregated, with the phenotypes above and muscular dystrophy as this is a clinical hallmark of recessive syndromes associated with the two other members of the POPDC family (i.e., *POPDC1* and *POPDC3*). We found a significant association with bradycardia after Bonferroni adjustment (*p* < 1.4 × 10^−3^) for the following variant sets: LOFCADD (*p* = 3.3 × 10^−4^, odds ratio [OR] 1.6, LOF and missense when predicted deleterious with CADD phred score ≥25) and LOF1MISID (*p* = 2 × 10^−4^, OR 1.59, LOF and missense when predicted deleterious by at least one of the following prediction methods: MetaSVM, MetaLR, or CADD phred score ≥25; Table S7). Although no association with a clinically relevant phenotype was detected, an association with slow heart rate was seen in heterozygous carriers in the general population. Whether this has clinical relevance remains to be explored.

## Discussion

We identified bi-allelic variants in *POPDC2* in four families that presented with a phenotypic spectrum consisting of sinus node dysfunction, AV conduction defects, and HCM. Using homology modeling, we show that the identified *POPDC2* variants are predicted to diminish the ability of POPDC2 to bind cAMP. In *in vitro* electrophysiological studies, we demonstrated that, while co-expression of WT POPDC2 with TREK-1 increased TREK-1 current density, POPDC2 harboring variants found in the affected individuals failed to increase TREK-1 current density. scRNA-seq from human hearts demonstrated that co-expression of *POPDC1* and -*2* was most prevalent in AV node, AV node pacemaker, and AV bundle cells. Sinus node cells expressed *POPDC2* abundantly, but expression of *POPDC1* was sparse. Together, these results concur with predisposition to AV node disease in humans with LOF variants in *POPDC1* and *POPDC2* and presence of sinus node disease in *POPDC2* but not in *POPDC1*-related disease in human. Our findings provide evidence for bi-allelic variants in *POPDC2* as the cause of a Mendelian autosomal recessive cardiac syndrome.

Several observations support the causality of the identified *POPDC2* variants (encoding p.Gln172_Tyr176delinsHis, p.Arg263His, p.Arg263Cys, p.Trp188Ter, and p.Leu37Serfs^∗^20). (1) The cardiac arrhythmia phenotype of the affected individuals harboring these variants in the homozygous or compound heterozygous state is similar to observations of sinus pauses and bradycardia that were made in mice lacking *Popdc2*^5^ and to AV block observed in zebrafish after morpholino knockdown of *popdc2*.^6^ (2) The variants affect highly conserved residues and are predicted damaging by multiple *in silico* prediction tools. (3) The variants are extremely rare in gnomAD^12^ and were not found in homozygous state. (4) A change affecting the paralogous residue of Arg263 (families B and C) in POPDC3, p.Arg261Gln, has been associated with muscular dystrophy (MIM: 605824).^28^ The dimeric homology model of POPDC2 predicted all disease variants we identified in the homozygous or compound heterozygous state to critically affect cAMP binding. One variant leads to the substitution of five residues with a single histidine residue (p.Gln172_Tyr176delinsHis; Figure 2B), and another two (p.Trp188Ter/p.Leu37Serfs^∗^20; Figure 2C) are expected to produce a truncated protein, all of which are expected to critically affect the ability of POPDC2 to bind cAMP.^25^ In addition, both transcripts could be subject to nonsense-mediated decay, which would result in no protein product being made. While the p.Arg263His and p.Arg263Cys variants are not predicted to disrupt protein folding, they are expected to eliminate a key interaction predicted to stabilize cAMP binding.^5^ Based on these predictions, we hypothesize that cAMP binding is critical for POPDC2 function and, when affected, causes disease.

Although heterozygous *POPDC2* variants have been proposed to increase susceptibility for cardiac conduction disease in humans by Rinné et al.,^35^ no Mendelian recessive disorder has been linked to this gene so far. In the study by Rinné et al.,^35^ a heterozygous nonsense variant in *POPDC2* (p.Trp188Ter; rs144241265, allele frequency of 1.70 × 10^−4^ among European individuals in gnomAD^12^) was identified in the heterozygous state in a monozygotic twin pair presenting with AV block and in an unrelated family with a mother and son both diagnosed with first-degree AV block (i.e., prolongation of the PR-interval on the ECG). The twin pair inherited the variant from the unaffected mother, indicating that it does not fully explain the phenotype observed in the two siblings. In support of this, single-variant analysis that we conducted in 162 carriers of the p.Trp188Ter variant in more than 1 million individuals from the general population did not show an association with sinus node or AV node conduction disease in heterozygous state. Thus, while the findings from Rinné et al.^35^ are interesting and support our findings, they do not establish pathogenic genetic variation in *POPDC2* as a (recessive or dominant) Mendelian cause of cardiac conduction disease in human. Based on our findings, we recommend the inclusion of *POPDC2* in clinical genetic-testing panels for affected individuals presenting with unexplained sinus node dysfunction, AV conduction defects with or without HCM. Using population-level genetic data of more than 1 million individuals, we showed that none of the variants were associated with clinical outcomes in heterozygous state, suggesting that heterozygous family members are unlikely to develop clinical manifestations and therefore might not necessitate clinical follow-up.

Bi-allelic variants in *POPDC1* and *POPDC3* have been associated with muscular dystrophy, with and without CCD, respectively,^4^^,^^5^ while the affected individuals reported here presented with isolated cardiac disease. Specifically, none of the affected individuals reported muscular weakness, atrophy, or cramps. Furthermore, muscle biopsy in the proband from family A did not show evident signs of muscle disease. Also, in contrast to *POPDC1*- and *PODPC3*-affected individuals, normal serum creatine kinase levels were found in the affected individuals reported here. While we did not detect a muscular phenotype in the affected individuals, currently aged 22–50 years, we cannot exclude subtle or age-dependent expression of muscular defects in *POPDC2*-related disease. Although all three POPDC proteins are expressed in both skeletal and cardiac muscle, differences in levels of expression between the POPDC proteins might, in part, determine phenotypic expression. Indeed, POPDC2 is predominantly expressed in cardiac tissue, whereas POPDC3, which presents with isolated muscular dystrophy, has a predominant expression in skeletal muscle.^36^

Western blot analysis and immunostaining of POPDC1 and POPDC2 in muscle biopsies obtained in the proband from family A showed significant reduction of the expression of both POPDC1 and POPDC2. These findings are in line with previous studies that suggested that stability and/or membrane trafficking of the POPDC1-POPDC2 complex is impaired by variants in each of the two proteins.^3^^,^^33^ Using spatial transcriptomics and scRNA-seq from human hearts, we showed that co-expression of *POPDC1* and *2* was most prevalent in AV node, AV node pacemaker, and AV bundle cells. On the other hand, in the sinus node, *POPDC2* was abundantly expressed, but expression of *POPDC1* was sparse. While these results support the observed predisposition to AV node disease in affected individuals with *POPDC2* LOF variants, proper statistical testing using pseudo-bulk counts was not possible due to a low number of donors with conduction-system data. Therefore, these results should be treated as hypothesis generating. Together, these results concur with predisposition to AV node disease in humans with LOF variants in *POPDC1* and POPDC2 and presence of sinus node disease in *POPDC2*- but not in *POPDC1*-related disease in human.

POPDC proteins are established interacting partners of the potassium channel TREK-1, which is known to underlie a background potassium current and is highly expressed in the sinus node.^30^ Co-expression of TREK-1 with any of the three POPDC proteins leads to an increase in TREK-1 current, a process modulated by the level of cAMP.^3^^,^^5^ Furthermore, cardiac-specific TREK-1-deficient mice display a sinus node phenotype characterized by bradycardia with frequent episodes of sinus pauses, partially resembling the phenotype in the affected individuals with *POPDC2* variants presented here.^30^ In an effort to shed light on the electrophysiological mechanism by which the variants in POPDC2 lead to bradycardia, we therefore conducted co-expression studies of WT and mutant POPDC2 with TREK-1. *In vitro*, both *POPDC2* variants tested failed to increase TREK-1 current and, by virtue of observations of bradycardia in TREK-1-deficient mice, these variants would be expected to be associated with bradycardia.

Notwithstanding the clear observation of bradycardia in TREK-1-deficient mice, how loss of background potassium current, causing an increase in diastolic net inward current, leads to bradycardia and sinus pauses is unclear. Our *in silico* modeling studies showed that a 41% reduction of TREK-1 current, simulating the effect of the variants in *POPDC2*, leads to an increase in diastolic depolarization rate and spontaneous firing. These findings are in agreement with experiments using isolated sinus node cells of TREK-1-deficient mice and computer simulations using a rabbit sinus node cell model.^30^ In a murine cardiac muscle cell line (HL-1), a stop variant in *Popdc2* associated with TREK-1 reduction, the maximum diastolic potential (MDP) was depolarized and the action potential upstroke velocity was reduced.^35^ In addition, a slower spontaneous firing rate was observed.^35^ In contrast, another study that examined loss of TREK-1 channel function in HL-1 cells showed an increase in spontaneous firing rate.^37^ Bradycardia may also be induced via changes in excitability of atrial cardiomyocytes surrounding the sinus node. In our simulated human atrial cell, reduction in TREK-1 current density slightly depolarized the MDP and increased the APD, consistent with findings in rat ventricular myocytes,^38^ but the excitability was hardly affected.

Thus, while collectively these data support a role for TREK-1 in cardiac pacing and a causal effect of *POPDC2* variants through modulation of TREK-1 current, the exact cellular electrophysiological mechanism remains unclear. Differences in the cellular models used cannot be excluded. Furthermore, the differences in effects of TREK-1 deficiency observed *in vivo* and *in vitro* in TREK-1-deficient mice suggest that other factors (such as altered sympathetic or parasympathetic stimulation *in vivo*) also contribute to the bradycardia observed at baseline in these mice.^30^ While an effect through modulation of sodium channel function could be postulated, no such effect was observed in our patch-clamp studies.

Although cardiac arrhythmias in affected individuals with recessive *POPDC2* variants are consistent with observations in mice^5^ and zebrafish,^6^ the role of *POPDC2* in cardiac hypertrophy remains unexplained. One of the potential mechanisms underlying cardiac hypertrophy in affected individuals with recessive variants in *POPDC2* is modulation of TREK-1. Since TREK-1 is activated by biomechanical stretch, the role of TREK-1 in cardiac responses to chronic pressure was recently studied using transverse aortic constriction.^39^ Notably, while no clear structural cardiac differences were seen at baseline, mice lacking TREK-1 exhibited an exaggerated pressure-overload-induced concentric hypertrophy with preserved systolic and diastolic cardiac function compared to WT mice.^39^

Affected individual 6 from family D (II-1 in Figure 1A) was diagnosed with bradycardia resulting in an arrest and first-degree AV block during an episode of fulminant myocarditis. We therefore cannot exclude a causal role of myocarditis in this case. However, (1) the conduction phenotype fits with the phenotypic characteristic of the other five affected individuals we report here; (2) the affected individual carried bi-allelic truncating variants in *POPDC2* likely to result in complete LOF, which is a known mechanism for disease in animal models; (3) there was no individual among 125,748 exomes and 15,708 genomes in gnomAD that carried both variants found in compound heterozygosity (p.Trp188Ter and p.Leu37Serfs^∗^20) in the affected individual from family D; (4) gnomAD contains 70 predicted LOF *POPDC2* variants and none of them occurs in the homozygous state; and (5) knockin mice of the p.Trp188Ter variant (found in compound heterozygosity in family D) displayed stress-induced sinus bradycardia and pauses.^35^ In aggregate, these data suggest a causal role for these variants in this individual. While at this stage speculative, some of the genetic cardiomyopathies (in particular in *DSP*) are associated with intermittent myocardial inflammatory episodes that appear clinically similar to myocarditis or sarcoidosis.^40^ Presentation with (recurrent) myocarditis-like episodes has been reported for arrhythmogenic cardiomyopathy (ACM).^41^ Among 560 probands and family members with ACM, Bariani et al. reported an episode resembling myocarditis (i.e., “hot phase”) in 23 cases (5%), particularly in pediatric affected individuals and carriers of desmoplakin (MIM: 125647) variants.^42^ Furthermore, in a population-based cohort of 336 consecutive affected individuals with acute myocarditis, a significant enrichment of pathogenic variants in genes associated with dilated or arrhythmogenic cardiomyopathy was found (8%) in comparison with controls (<1%, *p* = 0.0097).^40^ The question remains whether the myocarditis exposed the underlying *POPDC2*-related conduction disease or whether myocarditis is part of the *POPDC2* phenotypic spectrum. While we find it unlikely, compound heterozygosity of the truncating *POPDC2* variants might have been irrelevant in this case, and the phenotype could be fully the consequence of myocarditis.

This study is limited by its relatively small sample size, with four families presenting bi-allelic *POPDC2* variants, which might have not allowed us to provide the full clinical spectrum of disease associated with recessive *POPDC2* variants. Finally, while population-level data suggest no clinical manifestations in heterozygous carriers, longer-term clinical follow-up would be needed to confirm this in diverse populations and the heterozygous family members in *POPDC2* families.

We here provide robust association of bi-allelic variants in *POPDC2* with a Mendelian autosomal recessive cardiac syndrome consisting of sinus node dysfunction, AV conduction defects, and HCM. Future studies will help to illuminate the full clinical spectrum of the disease in individuals with bi-allelic variants as well as the clinical presentation of heterozygous carriers, thus elucidating the underlying mechanism of disease.

## Data and code availability

The genetic data from families A–D supporting the current study have not been deposited in a public repository because of ethical restrictions but are available from the corresponding author on request. The PDB coordinates for the POPDC2 homology models are available in supplemental information.

## Acknowledgments

We thank the families for their participation and collaboration. N.L. is supported by the 10.13039/501100003246Dutch Research Council (ZonMW VENI and Off-road), The Auxilium & Caritas Tulips Fellowship, and the De Snoo van 't Hoogerhuijs Award. C.R.B., A.V.P., and N.L. acknowledge the support from the 10.13039/100002129Dutch Heart Foundation (CVON 2018-30 PREDICT2 and CVON2014-18 CONCOR-GENES to C.R.B.) and the 10.13039/501100003246Netherlands Organisation for Scientific Research (VICI fellowship, 016.150.610, to C.R.B.). This work was supported in part by the 10.13039/100000002NIH awards R35GM128666 (M.V.A.) and T32GM092714 (F.Z.B.), a Sloan Research Fellowship (M.V.A.), and an American Heart Association Fellowship 23PRE1019634 (L.W.). This study makes use of data generated by the DECIPHER community. A full list of centers that contributed to the generation of the data is available from http://decipher.sanger.ac.uk and via email from decipher@sanger.ac.uk. Funding for the project was provided by the 10.13039/100010269Wellcome Trust.^7^ Website: https://www.deciphergenomics.org/. This project has been made possible in part by the Chan Zuckerberg Foundation (2019-202666) to M. Noseda and the British Heart Foundation and Deutsches Zentrum fur Herz-Kreislauf-Forschung (BHF/DZHK: SP/19/1/34461) to M.Noseda. M. Noseda and L. Mach were supported by the Rosetrees Trust Intermediate Project Grant (PGS23/100028) British Heart Foundation Centre of Research Excellence (RE/24/130023) and NIHR Imperial B­­iomedical Research Centre. L. Mach was further supported by British Heart Foundation Clinical Research Training Fellowship (FS/CRTF/23/24444), British Society for Heart Failure Research Fellowship, and Alexander Jansons Myocarditis UK. We thank Drs. Delphine Bichet and Florian Lesage (Universite de Nice Sophia Antipolis, France) for sharing the hTREK-1a plasmid. We thank Drs. Mohamed Hosny and Magdi Yacoub (Magdi Yacoub Foundation, Egypt) for reviewing the phenotype of affected individuals recruited at their center. R.T. is supported by the Canada Research Chairs program and the Philippa and Marvin Carsley Chair in cardiovascular genetics. The PNC “Hub Life Science- Diagnostica Avanzata (HLS-DA), PNC-E3-2022-23683266– CUP: C43C22001630001” is funded by the Italian Minister of Health. The support of Italian Ministry of Education and Research (MUR) “Dipartimenti di Eccellenza Program 2023–2027” - Dept of Pathophysiology and Transplantation, University of Milan to D.R. and G.P.C. is gratefully acknowledged. This work was promoted within the European Reference Network (ERN) for Rare Neuromuscular Diseases. We thank Sara Teichmann for sharing data on scRNA-seq in human hearts. The graphical abstract was created in https://BioRender.com.

## Author contributions

Conceptualization, N.L. and C.R.B.; data curation, N.L., M. Nicastro, A.M.C.V. P.G., R.T., A.V.P., E.M.L. P.A.Z., M. Alders, M. Allouba, Y.A., L. Mach, K.K., J.G., S.R., H.B., H.U., C.E., B.A., M.T.B., M.C., H.K.J., D.A.C, C.T., K.F., P.M.T., K.B., T.M.K., L.S., B.G.W., J.M., F.F., G.P., D.R., J.P.T., M. Noseda, M.A.V., I.C., A.A.M.W., R.W., and S.A.C.; investigation, N.L., M. Nicastro, F.Z.B., L.W., V.T., L. Mach, K.D., R.Z., L. Monserrat, J.L.S., D.U., A.M.A.M, K.K., S.J.J, J.C., I.E.Z., E.M.A.A., M.R., G.S., E.V.I., H.H., D.F.G., A.T.S., K.S., L.N., K.U.K., S.R.O., E.S., O.B.V.R., F.F., G.P., D.R., J.P.T., R.W., and A.O.V; visualization, N.L., M. Nicastro, F.Z.B., L.W., L. Mach, K.D., K.K., M.R., S.Z., and R.W.; writing – original draft, N.L., M. Nicastro, C.R.B., R.W., and A.O.V.; project administration, N.L., C.R.B., P.G.P., and L.B.; writing – review & editing, N.L., M. Nicastro, R.W., A.O.V., and C.R.B.; supervision, N.L. All authors read, revised, and approved the final manuscript.

## Declaration of interests

L. Monserrat is a shareholder in Dilemma Solutions SL. D.A.C. is an employee of and may own stock in GeneDx. H.M.A., V.T., G.S., E.V.I., H.H., D.F.G., A.T.S., and K.S. report employment at deCODE Genetics during the conduct of the study. C.E. reports grants from Abbott Diagnostics and Novo Nordisk outside the submitted work. K.U.K. reports research support from Intermountain Foundation during the conduct of the study. L.N. reports a stock option grant from Culmination Bio. H.B. reports lecture fees from Amgen, MSD, Sanofi Avensis, Bristol Myers Squibb, and Pfizer; grants from Novo Nordic Foundation; and another from Novo Nordic Foundation (shares) outside the submitted work.

## Supplemental information

Document S1. Figures S1–S6 and Notes S1 and S2

Data S1. Tables S1–S7

Data S2. POPDC2 dimer cAMP

Document S2. Article plus supplemental information
